# Metabolism Regulation and Redox State: Insight into the Role of Superoxide Dismutase 1

**DOI:** 10.3390/ijms21186606

**Published:** 2020-09-10

**Authors:** Simona Damiano, Concetta Sozio, Giuliana La Rosa, Bruna Guida, Raffaella Faraonio, Mariarosaria Santillo, Paolo Mondola

**Affiliations:** 1 Dipartimento di Medicina Clinica e Chirurgia, Università di Napoli “Federico II”, 80131 Naples, Italy; simona.damiano@unina.it (S.D.); sozioimma@gmail.com (C.S.); giuli_la-ro@libero.it (G.L.R.); bguida@unina.it (B.G.); 2 Dipartimento di Medicina Molecolare e Biotecnologie Mediche, Università di Napoli “Federico II”, 80131 Naples, Italy; raffaella.faraonio@unina.it

**Keywords:** superoxide dismutase 1, reactive oxygen species, metabolism, redox signaling, amyotrophic lateral sclerosis, cholesterol metabolism, mitochondria

## Abstract

Energy metabolism and redox state are strictly linked; energy metabolism is a source of reactive oxygen species (ROS) that, in turn, regulate the flux of metabolic pathways. Moreover, to assure redox homeostasis, metabolic pathways and antioxidant systems are often coordinately regulated. Several findings show that superoxide dismutase 1 (SOD1) enzyme has effects that go beyond its superoxide dismutase activity and that its functions are not limited to the intracellular compartment. Indeed, SOD1 is secreted through unconventional secretory pathways, carries out paracrine functions and circulates in the blood bound to lipoproteins. Striking experimental evidence links SOD1 to the redox regulation of metabolism. Important clues are provided by the systemic effects on energy metabolism observed in mutant SOD1-mediated amyotrophic lateral sclerosis (ALS). The purpose of this review is to analyze in detail the involvement of SOD1 in redox regulation of metabolism, nutrient sensing, cholesterol metabolism and regulation of mitochondrial respiration. The scientific literature on the relationship between ALS, mutated SOD1 and metabolism will also be explored, in order to highlight the metabolic functions of SOD1 whose biological role still presents numerous unexplored aspects that deserve further investigation.

## 1. Redox Regulation of Metabolism

### 1.1. Cellular Sources of ROS and Antioxidant Systems

As a result of normal cellular metabolism, cells continuously produce several types of reactive oxygen species (ROS) including superoxide anions, hydrogen peroxide, hydroxyl radicals, and a variety of their reaction products like organic hydroperoxides and hypochlorous acid [1].

ROS carry out important roles in both physiological and pathological conditions because of the opposite effects exerted by these highly reactive species; they can act as good or bad molecules, depending on the magnitude, duration and localization of their intracellular site of generation.

It is well known that many physiological functions are associated with the constitutive production of controlled ROS levels. Low or moderate amounts of oxygen radicals, in particular hydrogen peroxide (H_2_O_2_), carry out important roles as signal molecules that can modulate growth, hormone activity, synaptic transduction mechanisms, transcription factor activities, and other functions including food intake and energy metabolism [2,3,4,5,6]. On the other hand, altered cellular redox balance with elevated and/or protracted persistence of ROS has been linked to the pathogenesis of many human diseases, including premature aging, cancer, chronic inflammation, diabetes, ischemia/reperfusion injury, neurological disorders (e.g., amyotrophic lateral sclerosis, multiple sclerosis and Parkinson’s disease), age-related diseases and traumatic brain injury [7,8,9,10].

Cellular metabolism generates ATP through mitochondrial electron transport chain (ETC). During these reactions, small amounts of oxygen superoxide radical (O_2_^●−^), the principal ROS formed in mitochondria, are physiologically produced by addition of one electron to molecular oxygen. Superoxide is also formed during reverse electron transport (RET) from reduced coenzyme Q (CoQH2) to Complex I [11], and by other mitochondrial enzymes, in particular flavoenzymes [12]. Numerous reports highlight the importance of mitochondrial ROS in a variety of biological systems [13] including adaptation to hypoxia, regulation of autophagy, immunity, differentiation, and senescence, mainly functioning as signaling molecules facilitating cellular response to stress [14]. 

Increased O_2_^●−^ concentrations, observed in pathological conditions, leads also to peroxynitrite formation [15,16,17,18]. The peroxynitrite-derived radical species are involved in oxidation, peroxidation and nitration reactions with mitochondrial components [19,20].

In addition to mitochondria, relevant amount of ROS are generated by membrane-bound NADPH oxidase enzymes (NOX) that produce oxygen radicals through one electron reduction of molecular oxygen using NAD(P)H molecules as electron donors [21]. The NADPH oxidase enzyme family has seven members, five NOXs (NOX1–5), and two homologues, the dual oxidases (DUOX) 1 and 2. The latter, beside NADPH oxidase, are provided with an internal dismutase activity and release H_2_O_2_ as a product [22]. DUOX1/2 enzymes, first discovered in the thyroid where they play an essential physiological role in thyroid hormone synthesis [23], are also involved in innate immunity and cell signaling [24,25,26,27,28,29]. NOX-generated ROS modulate other physiological functions such as cell growth and differentiation as well as mucin expression and secretion [29,30,31]. The NADPH oxidases are also expressed in cells of the cardiovascular system and are involved in the development of hypertension [32]. 

Others sources of physiological ROS include different metabolic reactions catalyzed by various enzymes such as cyclooxigenase, lypooxigenase, xanthine oxidase and others [33].

Regarding the effects of ROS, it should be underlined that O_2_^●−^ is an instable and not easily diffusible molecule, whereas H_2_O_2_ is relatively more stable and readily diffuses across membranes to initiate downstream effects. Thus, H_2_O_2_ could serve as effective messenger to carry redox signals from generation sites to target sites [34]. Currently it has been ascertained that cell membranes present variable permeability to H_2_O_2_ due to their different lipid composition and to the presence of diffusion-facilitating channels that can be subjected to tight regulation [35]. Therefore, it is reasonable to speculate that the actions of these redox metabolites are largely limited in the cell compartment where they are produced, mainly in mitochondria and cytosol. Because H_2_O_2_ is designated to be the major redox signaling molecule, it is more dangerous than O_2_^●−^ when produced in an excessive/non-controlled amount. For these reasons, the physiological and pathological effects of these molecules depend on the amount and on their intracellular site of generation [36].

To defend against oxidative stress, cells are equipped with a variety of integrated enzymatic and nonenzymatic antioxidant systems. The superoxide dismutase (SODs) family represents the main class of antioxidant enzymes. In eukaryotic cells three forms of SODs are present: the dimeric cytosolic copper zinc superoxide dismutase (Cu, ZnSOD or SOD1) [37], the mitochondrial manganese superoxide dismutase (MnSOD or SOD2) [38] and the extracellular Cu, ZnSOD (SOD3) [39]. SOD1, the major superoxide dismutase isozyme, is ubiquitously expressed and is localized in the cytosol and in the intermembrane space of the mitochondria [40] as well as in the nucleus [41]. In contrast, SOD2 localizes exclusively in the mitochondrial matrix [42]. 

In addition to SOD family, other antioxidant enzymes are represented by catalase, and by numerous peroxidases (Px) or reductases based on the glutathione (GSH) system. Catalase reduce H_2_O_2_ to water and molecular oxygen, while GSH-Px and GSH-Reductase reduce H_2_O_2_ to water and/or lipid peroxides to their corresponding alcohols, at the expense of this low-molecular-weight thiol [43].

### 1.2. Impact of Nutrients on ROS Metabolism

Dietary macronutrients are organic compounds finalized to give energy for basal and energetic metabolism; however, they also act as key chemical signals inasmuch as they enormously affect ROS generation.

In eukaryotic cells, the metabolic pathways for energy production involve carbohydrate metabolism, mainly aerobic glycolysis, and fatty acid β-oxidation, that provides Acetyl CoA to the tricarboxylic acid (TCA) cycle for final oxidation. Such processes share the ability to form reduced electron carrier molecules (NADH and FADH_2_) that enter the ETC. The ETC is hosted in the inner membrane of mitochondria, where redox-active complexes and ubiquinone transfer electrons from NADH and FADH_2_ to molecular O_2_. Concomitant reactions generate a proton-motive force across the mitochondrial membrane that drives coordinated ATP synthesis; this process is referred to as oxidative phosphorylation (OXYPHOS). As a consequence of electron transfer at multiple sites, mitochondria are particularly suitable for producing basal amounts of superoxide anion, and they represent the major intracellular source of endogenous ROS (see Section 1.1). This also indicates that ROS production is significantly modulated by the amounts as well as the types of dietary nutrients. Thus, the production of ROS is strongly linked to energy metabolism and ROS in turn affect the redox status of many target proteins, including enzymes involved in nutrient metabolism [44,45]. Even if the link between nutrient intake and ROS production has been well established, with an unquestionable role played by H_2_O_2_, how nutrient signaling is integrated with redox regulation of molecules is an emerging and interesting question not yet completely understood. In fact, the way by which nutrients, and consequently ROS byproducts, represent signals able to affect cellular functions seems to be mainly ascribed to modulation of the redox status of target proteins containing Tyr or Cys residues [28].

Cells have many mechanisms sensing the different types of nutrients, allowing us to adjust and reprogram biochemical pathways to utilize them. For example, nutrient deprivation induces a metabolic switch from glycolysis to oxidative phosphorylation, a more efficient process in terms of energy production [46,47,48] that, however, is associated with increased ROS generation [49]. Nevertheless, nutrient sensors, in addition to determining metabolic reprogramming, also activate mechanisms preventing oxidative stress due to endogenous ROS increase. A typical example of how energy-based signals are linked to nuclear response is provided by the Keap1-Nrf2-ARE system [50]. In Keap1, redox modification of cysteines enables Nrf2 nuclear localization to drive transcription of Antioxidant Response Element (ARE)-dependent genes [51,52] that are implicated in antioxidant programs as well as in lipid and glucose metabolism. Interestingly, *SOD1* is one of the numerous genes induced by the Nrf2-ARE pathway [53]. 

Of note, SIRT3, which protects cells from oxidative stress, is induced by calorie restriction in rodents [54,55]. SIRT3 is a mitochondrial sirtuin belonging to the sirtuin family of nicotinamide adenine dinucleotide (NAD^+^)-dependent deacylases whose activity is highly dependent on NAD^+^ and therefore on cellular metabolic status [56]. SIRT3, in addition to directly or indirectly activating antioxidant enzymes like SOD2 [57], protecting cells from oxidative stress, also activates several mitochondrial enzymes. This represents a clear example of how mitochondrial activity and mechanisms of defense from oxidative stress are modulated in parallel by changes in the metabolic status of cells.

Another notable nutrient sensor is AMP-activated protein kinase (AMPK) [58,59,60,61]. AMPK is mainly an energy sensor [62] activated by a low cellular energy status coupled to an increase of AMP/ATP or ADP/ATP ratio. Recently, it has been highlighted that AMPK also senses glucose availability regardless of variation in adenine nucleotides levels [63]. AMPK is a serine/threonine kinase which phosphorylates specific enzymes, restoring energy balance by activation of catabolic pathways that generate ATP and downregulation of anabolic pathways and other processes consuming ATP. In addition, AMPK stimulates mitochondrial biogenesis, and mitochondrial quality control through regulation of autophagy and mitophagy [64]. AMPK acts in opposition to another key nutrient sensor, the mechanistic target-of-rapamycin complex 1 (mTORC1) activated by increased nutrient availability, especially amino acids and growth factors as will be detailed below (see Section 2). 

In addition to nutrients, these sensors can also be activated by ROS which, in turn, induce adaptation to oxidative stress. Indeed, AMPK is regulated by exogenous H_2_O_2_ [65], even if it is still unclear whether AMPK is directly activated by a ROS-sensitive kinases [66,67] or whether exogenous H_2_O_2_ activates respiratory chain elements, leading to a secondary effect on AMPK through increased AMP/ATP ratio [68]. On the other end, AMPK, provides cells with antioxidant defenses through NADPH maintenance [69]. During energy stress, AMPK decreases NADPH consumption by inhibiting acetyl-CoA carboxylases (ACC1 and ACC2), specifically involved in fatty-acid synthesis. Indeed, the pentose phosphate pathway that generates NADPH is impaired under glucose depletion. At the same time, AMPK increases NADPH generation through malic enzyme and isocitrate dehydrogenase that use malate and citrate, respectively, provided by the TCA cycle. Thus, fatty acid oxidation by supporting TCA cycle also maintains the NADPH homeostasis [69].

Moreover, in mouse embryonic fibroblasts (MEFs), glucose starvation activates AMPK, and through peroxisome proliferator-activated receptor (PPAR) γ and coactivator 1-α (PGC-1α), induces the expression of several antioxidant genes, including *CAT*, *SOD2*, and *Ucp2* [70].

ROS production is also induced by caloric overload from high carbohydrate or high-fat diets, which cause an excess of mitochondrial substrates; as a consequence, electron transport chain activity and ROS production increase [71]. 

In conclusion, nutrient excess, as well as nutrient deprivation, induce an abnormal ROS production above the physiological threshold. The close relationship between nutrient intake and ROS production, in part explains the pathogenetic mechanisms of metabolic diseases such as obesity, metabolic syndrome, type 2 diabetes and even cancer [72,73] as well as the aging process in which the defenses of antioxidant mechanisms are less effective [74,75].

## 2. Superoxide Dismutase 1 and mTOR Signaling

### 2.1. mTOR Complexes

Regarding the mechanisms involved in the modulation of cell metabolism and redox homeostasis by dietary nutrients, mechanistic rapamycin target complexes mTORC1 and mTORC2 seems to exert a perspective relevant role. These complexes mainly control cell growth and metabolism; they share the catalytic subunit mTOR, a serine/threonine kinase, while they differ in their other components, mechanisms of regulation, functions, and sensitivity to rapamycin, an antibiotic/antifungin functioning as an immunosuppressant. Indeed, acute treatment with rapamycin inhibits mTORC1; on the contrary, only under prolonged exposure to rapamycin could mTORC2 complex assembly be disrupted [76]. 

mTOR complexes are stimulated by nutrients and growth factors shifting cell metabolism in favor of anabolic pathways, while they are inhibited during fasting and by intracellular and environmental stress, thus ensuring cell growth merely in favorable conditions. mTORC1 and mTORC2 are sensitive to distinct stimuli, as mTORC1 mainly responds to nutrients while mTORC2 is sensitive to growth factor via PI3K signaling [77].

mTORC1 is activated by stimuli that operate after feeding when pro-growth endocrine signals are active and sufficient energy and nutrient levels are guaranteed; on the contrary, it is inhibited during fasting to limit the use of energy resources. Insulin/insulin-like growth factor-1 (IGF-1) pathways are mTORC1 activators; however, in addition to glucose-dependent insulin release, mTORC1 activation is also induced by changes in amino acid concentrations after feeding. In addition, mTORC1 responds to stress such as low ATP levels, hypoxia, or DNA damage. For example, glucose deprivation activates the stress responsive metabolic regulator AMPK, which inhibits mTORC1 [66,78,79]. Moreover, the DNA damage-response pathway inhibits mTORC1 through the induction *of p53 target* genes [80].

mTORC1 activation leads to increased protein synthesis and suppression of protein catabolism [81] and facilitates the accumulation of triglycerides by promoting adipogenesis and lipogenesis and by decreasing catabolic processes such as lipolysis and β-oxidation through a complicated downstream kinase involvement [82]. mTORC1 also promotes the synthesis of nucleotides required in growing and proliferating cells [83].

At cellular level mTORC1 has been localized mainly in the lysosomes, even if a pool of mTORC1 has been detected at other subcellular sites including mitochondria, ribosomes, nucleus and lipid rafts. The different subcellular localizations may be very important for mTOR functions to enact precise spatial and temporal control of cell growth [84].

In contrast to mTORC1, mTORC2 primarily functions as an effector of insulin/PI3K signaling leading to the phosphorylation and activation of Akt [85]. The role of mTORC2 consists mainly of controlling growth by regulating lipogenesis, glucose metabolism [86,87], actin cytoskeleton [76,88], cell survival and apoptosis [89]. mTORC2 signaling pathway was also thought to regulate cytoskeleton organization by phosphorylation-activating protein kinase C (PKC)α, Akt, or serum- and glucocorticoid-induced protein kinase-1 (SGK1) [90]. Moreover, some evidence suggests that mTORC2 is essential for the regulation of neuronal morphology and synaptic activity [91].

### 2.2. mTOR in the Hypothalamic Control of Food Intake and Energy Balance

The hypothalamus receives nutrients and hormone signals coming from peripheric tissues that modulate the activity of two populations of neurons in the arcuate nucleus (ARC): orexigenic neurons expressing both neurotransmitters neuropeptide Y (NPY) and agouti-related peptide (AgRP) and anorexigenic neurons coexpressing proopiomelanocortin (POMC), cocaine- and amphetamine-regulated transcript (CART). 

mTORC1 colocalizes with AgRP/NPY and POMC neurons in the ARC [92,93]. It has been well documented that its activity is associated with the regulation of food intake, body weight, energy expenditure, and glucose/lipid homeostasis even if, until now, the mechanisms underlined have not yet completely understood.

Fasting and refeeding reduce and increase, respectively, phosphorylation of mTORC1 in the rat medial-basal hypothalamus (MBH) [94], suggesting that hypothalamic mTORC1 activity is closely associated with the energy status of animals. 

The mTORC1 signaling pathway in the hypothalamus is regulated by nutrients, mainly amino acids and glucose. Amino acids such as leucine and arginine are potent activators of hypothalamic mTORC1, through interaction with Rag proteins, another set of small GTPases [95]. Interesting data show that rapamycin treatment, inhibiting mTORC1, increases the orexigenic Agrp mRNA levels in cells exposed to high amino acid concentration; these observations indicate that amino acids can act within the brain to inhibit food intake and that a direct, mTOR-dependent inhibition of *AGRP* gene expression may contribute to this effect [96].

Other data have shown that mTOR mediates the decrease of food intake and body weight in rats following central administration of leucine by decreasing the expression levels of Agrp and NPY and increasing POMC expression within the ARC [94,97]. Of note, it must be underlined that overnutrition impairs mTORC1 activity and decreases mTORC1 signaling in the hypothalamus; this effect contributes to the development of hyperphagia, weight gain, and leptin resistance in high-fat-diet (HFD)-induced obesity [98].

At the hypothalamic level, mTOR also integrates signals from a variety of hormonal stimuli such as leptin, insulin, and ghrelin, although its action varies in different neuronal populations [99].

Ghrelin and leptin exert opposite regulatory effects on feeding behavior and metabolism acting on POMC and AgRP neurons in the ARC; their effects are mediated by mTORC1 activity [90,94], suggesting that mTORC1 may serve as a switch mechanism able to mediate the diverse role of these two hormones in the regulation of food intake [93].

### 2.3. Modulation of SOD1 Activity by mTORC1

**Figure 1:**
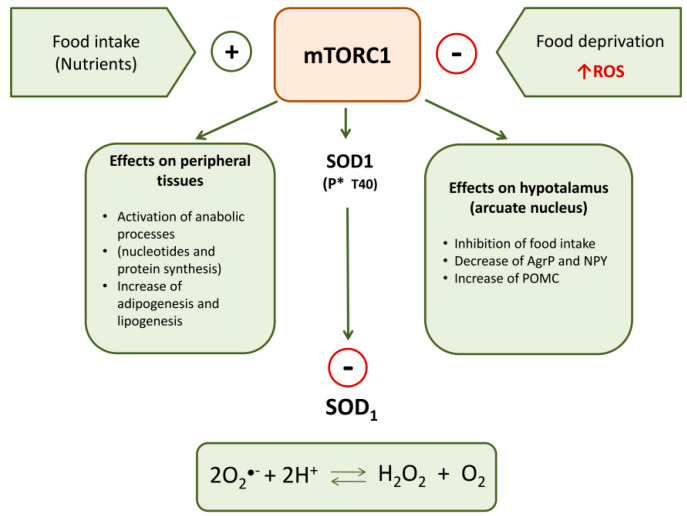
Principal peripheral and central effects of mTORC1 on metabolism and SOD1 activity.

Recently, Tsang et al. [100] reported a relation among SOD1, food intake-mediated ROS and mTORC1. SOD1 is a target of mTORC1 in nutrient signaling; in particular, mTORC1 regulates SOD1 activity through its reversible phosphorylation at threonine 40 (Thr-40). This kinase rapidly phosphorylates SOD1 in mammalian cells in response to nutrient signaling and this negatively influences the antioxidant activity of this enzyme. Therefore, nutrients stimulate mTORC1 that phosphorylates SOD1 inactivating its dismutase activity; in this way, nutrient signaling modulates ROS levels (Figure 1). During starvation, by the removal of mTORC1 inhibition of SOD1, the increase of ROS is counteracted, thus assuring protection against oxidative stress. Therefore, SOD1 phosphorylation by mTORC1 provides a dynamic mechanism in eukaryotic cells for redox control under varying nutrient conditions. It permits rapid growth in rich nutrient conditions while conferring resistance to O^●−^ radical-induced stress during starvation. 

## 3. SOD1, Diet and Cholesterol Homeostasis

### 3.1. SOD1 as Target of Dietary Interventions

Some dietary interventions induce an increase in *SOD1* gene expression. Foods with anti-inflammatory properties usually have antioxidant activities as well, and enhance intracellular enzymatic antioxidant systems. For instance, a hazelnut-enriched diet, in addition to exerting beneficial anti-inflammatory effects, also induces the expression of antioxidant enzymes including SOD1 [101]. Another example is given by anthocyanin-rich color wheat supplementation in mice fed with high-fat diets; by nutrigenomic approach it has been shown that this dietary intervention enhances fatty acid oxidation and reduces ROS by acting as an antioxidant itself and by inducing antioxidant enzymes like SOD1 [102]. Analogous effects on SOD1 expression are exerted in mice under ketogenic diet, a high-fat, low-carbohydrate and low-protein diet. A ketogenic diet mimics the metabolic effects of chronic starvation with a shift of energy substrate utilization from glucose towards fatty acids and consequently induction of oxidative stress [103]. 

### 3.2. Presence of SOD1 in Serum Lipoprotein

The presence of SOD1 in the blood has been for long time explained as deriving from physiological red cell hemolysis. However, the existence of SOD1 secretion by many cell lines changed this axiom, suggesting that serum SOD1 can derive, at least partially, from peripheral tissues secretion (see below).

It is known that circulating lipoproteins transport several substances such as albumin, neutral lipids, cholesterol, apoproteins that regulate the lipoprotein metabolism, and antioxidants as vitamins A and E, which carry out an important protective effect against lipoprotein oxidation. Low density lipoproteins (LDL) represent the principal form of cholesterol transport in humans and an increase of their serum concentration represents an important biochemical event leading to atherosclerosis whereas high levels of high density lipoprotein (HDL) cholesterol appears to be protective [104]; LDL oxidation, increasing the half-life of lipoproteins, is responsible for their accumulation in arterial walls [105]. The resistance of plasma LDL to oxidative processes is widely assumed to be a good protective indicator against the atherogenic risk. 

The analysis of SOD1 distribution and activity among different human lipoproteins [106] evidenced that SOD1 is noticeably present in all serum lipoprotein classes, mainly in LDL and HDL. SOD1 binds to the lipid component of lipoproteins as demonstrated by the fact that when SOD1 is incubated with lipid emulsion and further ultracentrifuged to separate the lipids from the aqueous phase, all the SOD1 is detected in the top lipid phase. SOD1 linked to lipoproteins could exert a physiological protective role against oxidative damages, avoiding lipoperoxidation that can extend the half-life of circulating lipoproteins, impairing their metabolism. Confirmation of the protective role of SOD1 against lipoprotein oxidation derived from gene expression profile analysis using cDNA microarray of isolated macrophages from atherosclerotic coronary plaque from hypercholesterolemic swine. This study showed that SOD1 displayed the strongest inverse correlation with oxidized LDL [107].

### 3.3. Effects of SOD1 on HMGCoA Reductase and LDL Receptor

The microsomal enzyme 3-hydroxy-3-methylglutaryl CoA (HMG-CoA) reductase and the LDL receptor pathway carry out a key role on cholesterol homeostasis in humans. Brown and Goldstein’s classical experiments [108,109] demonstrated that when intracellular cholesterol is too high, cells downregulate cholesterol synthesis and LDL cholesterol uptake. 

The HMG-CoA reductase is sensitive to oxidative inactivation and to phosphorylation by many kinases that inactivate this enzyme and increase its susceptibility to proteolysis [110].

The involvement of SOD1 in cholesterol metabolism is suggested by data [111] showing that in human hepatocarcinoma HepG2 cells, SOD1 is able to affect cholesterol metabolism, decreasing HMG-CoA reductase activity and its protein levels. This inhibitory effect was accompanied by reduced cholesterol synthesis measured as [^14^C]acetate incorporation into [^14^C]cholesterol and by an increased [^125^I]LDL binding to HepG2 cells; SOD1 effects are mediated by an activation of protein kinase C [111]. Most of the effects of SOD1 on cholesterol metabolism detailed above cannot be ascribed to its dismutase activity since they were also observed in cells treated with the metal-free, inactive SOD1 and with SOD1 inactivated with hydrogen peroxide, as well as in cells treated with the full active enzyme [111]. This is only an example of SOD1 effects independent of its antioxidant properties (see Section 7). 

**Figure 2:**
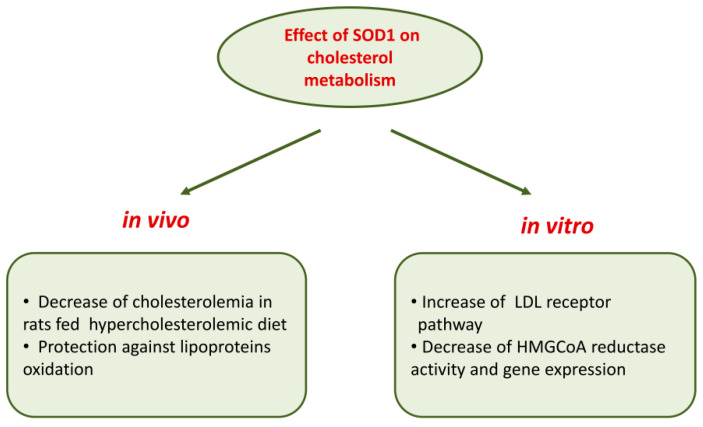
Effects of SOD1 on cholesterol metabolism.

Further studies performed in wild-type human fibroblasts, in hepatocarcinoma Hep G2 cells and in fibroblasts of subjects affected by familiar hypercholesterolemia demonstrated that SOD1 inhibited HMG-CoA reductase gene expression at the transcriptional level; in addition, a strong downregulation of gene expression of sterol regulatory element-binding proteins (SREBP-2), a membrane-bound transcriptional factor, and LDL receptor was observed [112]. Figure 2 recapitulates the overall effects of SOD1 on cholesterol metabolism. 

## 4. SOD1-Mediated Repression of Mitochondrial Respiration

Various highly proliferating cells such as cancer cells, lymphocytes, endothelial cells or yeast strains like Saccharomyces cerevisiae undertake glucose-mediated repression of respiration in favor of aerobic fermentation (i.e., fermentation which takes place in the presence of oxygen) with lactate or ethanol production; in this way more NAD^+^ is generated which allows glycolysis to continue [113]. This process consents proliferating cells to utilize nutrients more efficiently as building blocks for the biosynthetic pathways rather than for catabolic oxidation, conferring an advantage for cell growth. 

Different mechanisms contribute to the switch from respiration to aerobic fermentation. In yeasts, glucose activates a series of signaling pathways that repress respiration and promote aerobic fermentation [114]. 

**Figure 3:**
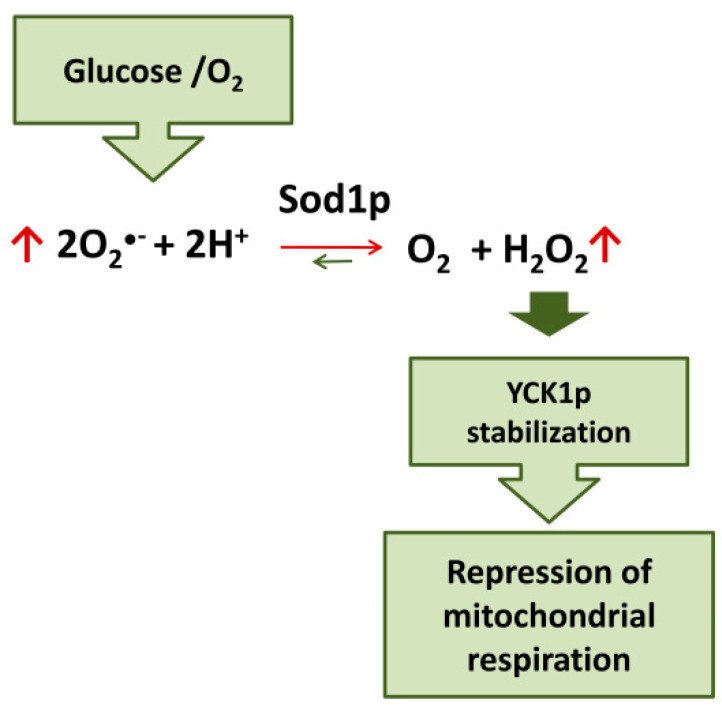
Sod1p mediates the repression of mitochondrial respiration by glucose/O_2_ in yeast.

Evidence on the role of SOD1 in glucose- and oxygen-mediated repression of respiration in yeast has recently been accumulated. Yeast strains lacking CuZn superoxide dismutase (Sod1p) fail to completely repress respiration in the presence of glucose [115]. Moreover, it has been demonstrated that the mechanism involved in glucose-mediated repression of respiration in yeast cells involves the Sod1p-stabilization of casein kinase 1-gamma (CK1γ) homologs, Yck1p and Yck2p; these kinases are essential for glucose sensing and activation of pathways downstream leading to respiration repression [116,117]. Sod1p physically interacts with Yck1p; superoxide produced in the presence of oxygen and glucose is transformed by Sod1p in H_2_O_2_ which stabilize Yck1p. Therefore, glucose and O_2_ stabilize these casein kinases seemingly by controlling the amount of superoxide substrate for Sod1p. In this way, SOD1 transmits signals from oxygen and glucose to repress respiration [118] (Figure 3). 

Mammalian SOD1, like its yeast counterpart, suppresses mitochondrial respiration; indeed, transfection of HEK 293 cells with human SOD1 decreases mitochondrial oxygen flux [119]. Therefore, it is likely that repression of respiration is a metabolic function of SOD1 conserved from yeast to humans. SOD1-mediated suppression of respiration is modulated by acetylation, a very common mechanism of metabolic regulation. In particular, lysine acetylation is a post-translational modification regulating several enzymes of intermediate metabolism [120].

By proteomic approach, it has been demonstrated the acylation of lysine 122 (K122) on SOD1; this post-translational modification suppressed the ability of SOD1 to inhibit mitochondrial respiration at respiratory complex I without impairing its enzymatic activity [119]. Increasing K122 acylation on SOD1 by depleting SIRT5 deacylase inhibits the anti-respiratory activity of SOD1. Moreover, transfection of HEK 293 cells with acetyl-mimicking (K122Q) mutant of SOD1, unlike the wild type form of the enzyme, did not affect mitochondrial respiration [119]. Acyl-mimicking mutations at K122 decreased SOD1 accumulation in mitochondria, but SOD1-mediated inhibition of respiration is upstream of its mitochondrial localization since K122 acyl mutants forced to reach the mitochondrial intermembrane space by an intermembrane-targeting tag are still unable to suppress respiration when expressed in HEK 293 cells [119]. Therefore, it seems that deacylation of SOD1 is essential for its effects on respiration, which in turn elevates levels of mitochondrial SOD1, thus reducing mitochondrial stress. The suppression of respiration by SOD1 in its deacetylated form and its mitochondria accumulation could be viewed as an additional mechanism contributing to the antioxidant/prosurvival function of this enzyme. 

SOD1 seems also involved in adipogenesis, a multistep process essential for metabolic homeostasis of the organism, allowing lipid storage and release, and avoiding ectopic accumulation of lipids in tissues and organs. An adipogenesis defect represents a hallmark of obesity, insulin resistance and aging [121,122,123]. Mice lacking SOD1 show reduced levels of adipogenic transcription factors, Cebpα and Pparγ, and of the master regulator of mitochondrial biogenesis, Pgc1α, compared to wild-type C57BL/6JRj mice [124].

## 5. Redox and Metabolic Dysregulation in Mutant SOD1 Linked Familial Amyotrophic Lateral Sclerosis

Amyotrophic lateral sclerosis (ALS) is an adult-onset neurodegenerative disease associated with a fatal loss of cerebral cortex, brain stem and spinal cord motoneurons finally progressing to muscle atrophy and paralysis [125]. Approximately 90% of ALS cases are sporadic (sALS), with the remaining 10% being inherited familial ALS (fALS). Among fALS, approximately 10 to 20% (1–2% of total cases) are associated with mutations in the gene encoding for SOD1 [126].

Abnormalities in ALS are not restricted to motoneurons but rather ALS can be considered a systemic disease with spreading effects including several defects in energy metabolism. Energy imbalance is associated with weight loss and hypermetabolism; ALS patients usually lose weight and body fat with disease progression [127]. In addition, hyperlipidemia with increased LDL-cholesterol and decreased HDL-cholesterol levels have been found in ALS patients in different studies [128]. Furthermore, multiple genetic analyses show that elevated low-density lipoprotein cholesterol is a causal risk factor for ALS [129]. The dysregulation of energy metabolism in ALS is also supported by studies in rodent models. In transgenic mice expressing human mutant SOD1, the most common genetic animal model of ALS, metabolism is higher and body weight and fat mass are lower compared to wild-type mice [130].

Among cellular and molecular mechanisms suggested to explain motoneuron degeneration in ALS, much attention has been paid to mitochondria-mediated damage. Mitochondrial defects and abnormalities in motoneurons of ALS patients and the SOD1 mouse model of ALS have been reported [131]. In the spinal cord of mutant SOD1 mice, mitochondria dysfunction occurs during the pre-symptomatic phase of disease [132,133,134,135,136,137] immediately before the onset of motoneuron degeneration [135]; this indicates that mitochondria dysfunction cannot be considered a secondary event associated with the disease state but rather is a key player in initiating the events leading to motoneuron loss in ALS. 

In mutant SOD1 mice, mitochondria Ca^2+^ buffering capacity and respiration were impaired [132,133,134,135,136,137]. A hallmark of ALS is the presence of aggregated proteins including SOD1 at the surface of outer mitochondrial membrane suggesting a direct impact of this enzyme on mitochondria functions [138,139,140]. Moreover, mutated SOD1 shows increased affinity for Bcl-2 [138] and this interaction could lead to a metabolic switch from mitochondrial respiration which is limited by Bcl-2/SOD1 interaction towards glycolysis [141]. Moreover, an activation of AMPK signaling was evidenced in motoneurons of ALS patients [142] and in embryonic neural stem cells derived from SOD1G93A mice [143]. AMPK is a central regulator of cellular metabolism implicated in multiple metabolic functions including glycolysis, lipid metabolism and mitochondrial function [64].

In ALS, defects in mitochondrial functions have also been found in cells other than motoneurons [132], including muscle cells and astrocytes. In the skeletal muscle of mutant SOD1 mice [144] and ALS patients [145] an upregulation of mitochondrial uncoupling protein 3 (UCP3) has been found. These findings can account, at least in part, for increased energy needs and hypermetabolism in ALS.

**Figure 4:**
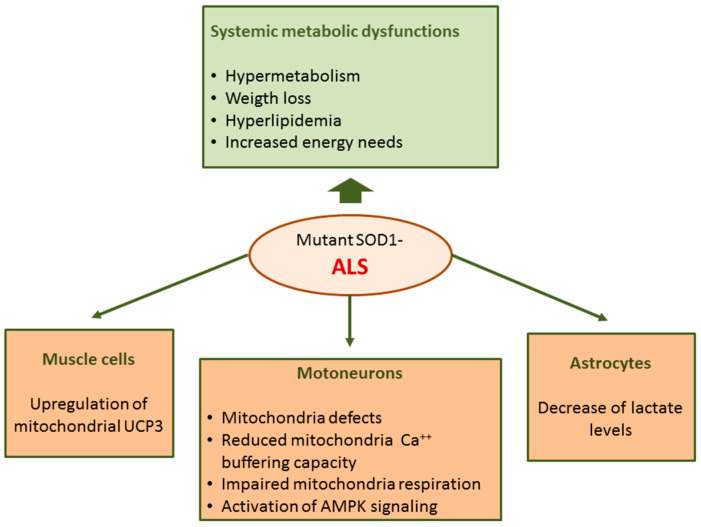
Metabolic dysregulation in mutant SOD1-linked familial amyotrophic lateral sclerosis (fALS).

Astrocyte and microglia activation is another pathological hallmark of the disease. Among other functions, astrocytes provide metabolic support to neurons. A metabolomics study performed on co-cultures primary astrocyte and motoneurons has evidenced that SOD1G93A mutation induces metabolic changes in astrocytes with a decrease in extra- and intra-cellular lactate levels [146]; these data suggest that the metabolic dysfunction of astrocytes in ALS could contribute to astrocyte-mediated neurotoxicity (Figure 4). 

Extensive crosstalk exists among the pathways regulating mitochondrial respiration, intracellular calcium and redox balance which are all altered in ALS, as discussed above. According to the recent homeostatic instability theory, formulated to explain the pathophysiological mechanisms of ALS, the impairment of these functions can be interpreted as failure of cellular regulatory and homeostatic control. Because of their particular properties, motoneurons seem particularly susceptible to homeostatic instability [147]. A systematic review analyzing experimental data from 45 studies revealed a failure of homeostatic regulation in ALS animal models, mainly SOD1G93A transgenic mice [148]. Analysis of overall trends showed that cellular respiration, ATP levels and other markers of mitochondrial activity are depressed before symptom onset, and remain at low levels throughout the entire course of disease; on the contrary, markers of oxidative stress are increased only by the onset of symptoms despite a post-natal early increase of heat shock proteins representing a compensatory response to oxidative stress [149]. Finally, intracellular calcium increased early and is then compensated at the pre-onset of symptomatology to increase again by post-onset. These data suggest that in ALS there exists an impairment of the compensatory mechanisms able to assure homeostasis. Further elements in favor of the homeostatic instability theory is given by the observation that ALS subjects have lower rates of antecedent diseases (hypertension, liver disease, hyperlipidemia, and others) than the general population [150]; moreover, a later onset of ALS in patients with antecedent diseases has been observed [151] and there is an inverse correlation between onset age and disease duration [152]. These findings led to the hypothesis that a too-high feedback gain of regulatory mechanisms (named hypervigilant regulation) correct small imbalances from homeostasis in asymptomatic ALS subjects protecting them from antecedent disease and delaying the age of ALS onset. However, the pathological overreaction of regulatory processes ultimately leads to ALS and to reduced patient survival [150,152].

## 6. SOD1 in T Cell Activation

Metabolic control of ROS production has long been recognized as a regulator of T cell activation. In fact, ROS act as intracellular signaling molecules modulating immune system functions either in steady-state or upon antigen recognition, influencing the outcome of the T cell response [153] and the development of autoimmune diseases [154]. Several studies show that T cell receptor (TCR)-dependent T cell activation induces ROS generation [155,156,157] by mitochondrial respiratory chain [14], lipoxygenases and NADPH oxidases [158,159]. Among ROS, H_2_O_2_ plays a major role as the second messenger in antigen receptor signaling [160,161,162]. Treatment of lymphocytes with H_2_O_2_ can mimic the effect of antigen exposure; H_2_O_2_ can directly oxidize receptor protein, or induce receptor cross-linking or conformational changes leading to its activation. In addition, H_2_O_2_ can activate intracellular protein tyrosine kinases downstream receptor activation or, more importantly, can inhibit protein tyrosine phosphatase [163]. A close relationship between T cell activation and SOD1 has been demonstrated since the activation of T lymphocytes is capable of inducing both intracellular increase and brefeldin (BFA)-dependent secretion of SOD1 and a cellular re-localization of the enzyme [164]. Indeed, TCR and SOD1 co-localize and cluster after TCR triggering in human T cells. Since H_2_O_2_ is the most relevant oxidant species that regulates TCR signaling, SOD1 intracellular re-localization upon TCR-triggering suggests that SOD1 could serve to increase H_2_O_2_ production ensuring the source of oxidants necessary to modulate kinase/phosphatase activity related to TCR signaling. Finally, an additional link between SOD1 and immune system functions comes from clinical studies on ALS. Immune dysregulation is an hallmark of mutant SOD1 ALS, even if enhanced neuroinflammation and dysfunctional regulatory T lymphocytes are observed in multiple genetic mutations linked to ALS, other than SOD1, as well as in the sporadic forms of ALS [165]. 

## 7. SOD1 Functions beyond Its Role as Superoxide Scavenger

It has been shown that SOD1 lacking a signal sequence for entering the conventional ER–Golgi complex pathway of secretion is constitutively exported by many cellular lines by unconventional secretion pathways [166,167,168,169,170]. Moreover, in excitable cells, in addition to basal SOD1 secretion, this enzyme is also exported following depolarization induced by high extracellular K^+^ concentration [5,171]. In NSC-34 motor neuron cell cultures expressing G93A SOD1 mutant, a cellular model of mutant SOD1-mediated ALS, an impairment of mutant SOD1 secretion related to neurotoxicity has been reported [170,172]. 

Recently, it has been shown that wild-type and ALS-linked mutant SOD1 undergo a nutrient starvation-specific unconventional secretion, like acyl-CoA binding protein 1 (Acb1) [173,174]. A conserved diacidic motif (Asp-Glu) at positions 77/78 was reported to be essential for the starvation-induced export of SOD1. The physiological significance of this event is not clear but it is possible to hypothesize that increased ROS levels under nutrient deficiency could determine an increase of the levels of oxidant molecules also in the extracellular space which can be protected, enhancing SOD1 secretion. 

The discovery of SOD1 export in the extracellular compartment and the observation that cellular SOD1 levels are far higher the amount needed to maintain ROS below cytotoxic levels [119] pioneered the discovery of inedited effects of this enzyme whose role is not limited to its superoxide dismutase activity. [175]. In fact, it has been demonstrated that in NSC-34 motor neurons and in human SK-N-BE neuroblastoma cells, SOD1 is able to interact with muscarinic M1 receptor activating phospholipase C signaling with consequent intracellular calcium increase. These effects were independent on dismutase activity of SOD1 since metal-free SOD1 is able to reproduce the effects of the active enzyme [176,177]. Moreover, experiments carried out in rat pituitary GH3 cells evidenced that SOD1 inhibits the P-ERK1/2 pathway through an interaction with muscarinic M1 receptors [178]. The paracrine role of SOD1 has been confirmed in in vivo studies. Intracerebral injection of SOD1 in the dentate gyrus of the rat hippocampus inhibits long term potentiation (LTP) induced by high frequency stimulation of the perforant path. Similar effects were observed when apo SOD1, the metal-free form of SOD1 lacking enzymatic activity, was administered to the animals, thus demonstrating that the effects of the full active enzyme can be only in part ascribed to the superoxide dismutase activity [179]. 

Another notable non canonical function of SOD1 is its function as a transcription factor regulating gene expression. Oxidative stress induces nuclear translocation of SOD1 through Sod1 phosphorylation at Serine60 and 99 mediated by Mec1/ATM and its effector Dun1/Cds1 kinase. In the nucleus, SOD1 activates genes involved in the response to oxidative stress, replication stress, DNA damage, and Cu/Fe homeostasis by directly binding to their promoters [180].

## 8. Concluding Remarks and Future Directions

Different nutrient conditions can have a significant impact on ROS production; moreover, increasing experimental evidence demonstrates that ROS influence the redox potential of many target proteins including enzymes involved in metabolism. Therefore, it is reasonable to assume that enzyme activity regulating the nutrient metabolism is affected by the modification of their redox status. 

Many data highlight the link between SOD1 and metabolism. Recent results demonstrated that reversible phosphorylation of SOD1 by mTORC1 inhibits superoxide dismutase activity of SOD1 enabling nutrient signaling to directly control the level of superoxide radicals. Other metabolic effects of SOD1 are respiration repression which could contribute together with dismutase activity to the antioxidant and pro-survival effects of SOD1. Further notable evidence of the link between SOD1 and metabolism emerge from the mutant SOD1-linked ALS where hypermetabolism, weight loss and body fat loss are hallmarks of the disease.

Moreover, in vitro and in vivo experiments have shown that SOD1 carries out a role on cholesterol metabolism either decreasing the HMGCoA reductase, the key enzyme of cholesterol synthesis, or increasing the LDL receptor pathway.

Despite the numerous findings linking SOD1 with metabolism, in most cases the mechanisms underlined remain elusive. From all these considerations it follows that it is prospectively interesting to further investigate the role of ROS and SOD1 in nutrient signaling and redox homeostasis in physiological and pathological conditions.

## Abbreviations

Acb1	Acyl-CoA binding protein
ACC1 and ACC2	Acetyl-CoA carboxylases
AgRP	Agouti-related peptide
Akt	Protein kinase B
ALS	Amyotrophic lateral sclerosis
AMPK	5′ adenosine monophosphate-activated protein kinase
ARC	Arcuate nucleus
Bcl-2	B-cell lymphoma 2
CART	Cocaine- and amphetamine-regulated transcript
Cebpα	Transcriptional Repressor of T-Cell
CK1γ	Casein kinase 1-gamma
CoQH2	Reduced coenzyme Q
DUOX	Dual oxidases
ERK1-2	extracellular signal-regulated kinase
ETC	Electron transport chain
fALS	Familial ALS
FFA	Free fatty acid
GSH-Px	Glutathione peroxidase
HDL	High density lipoproteins
HepG2	Human hepatocarcinoma cell line
HFD	High-fat-diet
HMGCoA	3-hydroxy-3-methylglutaryl-CoA
HMG-CoA	Microsomal enzyme 3-hydroxy-3-methylglutaryl CoA
IGF-1	Insulin/insulin-like growth factor-1
LDL	Low density lipoproteins
LTP	Long term potentiation
MBH	Medial-basal hypothalamus
MEFs	Mouse embryonic fibroblasts
MnSOD	Manganese superoxide dismutase
mTORC1	Mechanistic target-of-rapamycin complex 1
NOXs	NADPH oxidase enzymes
NPY	Neuropeptide Y
NSC-34	Mouse Motor Neuron-Like Hybrid Cell Line
OXYPHOS	Oxidative phosphorylation
PGC-1α	Peroxisome proliferative activated receptor, gamma, coactivator 1
PI3K	Phosphoinositide 3-kinases
PKC	Protein kinase C
POMC	Anorexigenic neurons coexpressing proopiomelanocortin
PPAR	Peroxisome proliferator-activated receptor
RET	Reverse electron transport
ROS	Reactive oxygen species
sALS	Sporadic ALS
SGK1	Serum-and glucocorticoid-induced protein kinase-1
SIRT3	Sirtuin3
SK-N-BE	Human neuroblastoma cell line
SOD	Superoxide dismutase
TCR	T cell receptor
UCP	Mitochondrial uncoupling protein
